# Quality of life and objective-subjective functionality in individuals with schizophrenia

**DOI:** 10.1192/j.eurpsy.2024.1502

**Published:** 2024-08-27

**Authors:** A. M. Lisincki, L. Csizmadia, F. Á. Szabó, J. Réthelyi, É. Jekkel

**Affiliations:** Department of Psychiatry and Psychotherapy, Semmelweis University, Budapest, Hungary

## Abstract

**Introduction:**

Chronic mental illnesses can significantly impact an individual’s quality of life and lead to functional disabilities. Scientific interest in overall quality of life and health-related quality of life has been gradually increasing, especially in the context of chronic diseases where the relationship between patients’ long-term functionality and symptom development is not always linear.

**Objectives:**

Our research aimed to investigate the factors influencing subjective-objective well-being and in patients diagnosed with schizophrenia and schizoaffective disorder. Specifically, we examined the effects of anticipated discrimination on patients’ quality of life, satisfaction with health care, and overall functionality.

**Methods:**

We recruited 25 patients from Semmelweis University Department of Psychiatry and Psychotherapy in Budapest, Hungary. To be eligible, patients had to meet the diagnostic criteria for schizophrenia or schizoaffective disorder according to DSM-5, cooperate with pharmacotherapy, and meet remission criteria (Andreasen et al., Am J Psychiatry 2005; 162 441-449). We collected socio-demographic data and clinical history, utilized the Mini International Neuropsychiatric Interview (M.I.N.I.) and the Positive and Negative Syndrome Scale (PANSS) to identify our clinical sample and assess the severity of symptoms. Objective and subjective functionality and well-being were measured using the Lancashire Quality of Life Profile (LQoLP). Self-reported medication adherence were measured with Morisky Medication Adherence Scale (MMAS-8). Additionally, we assessed anticipated discrimination (QUAD), and satisfaction with healthcare (CACHE).

**Results:**

Our findings have unveiled a cross-sectional association between higher self-reported medication adherence and improved quality of life among patients with schizophrenia. Moreover, increased adherence levels, as well as greater satisfaction with healthcare, were linked to enhanced objective and subjective functionality and overall well-being. Additionally, the anticipation of discrimination was found to be associated with reduced quality of life and functionality.

**Conclusions:**

The overall quality of life and objective-subjective functioning in patients diagnosed with schizophrenia and schizoaffective disorder can be influenced by various factors. Further research is needed to gain a better understanding of the factors associated with higher quality of life in patients.

**Disclosure of Interest:**

None Declared

